# Therapeutic targeting of JAKs: from hematology to rheumatology and from the first to the second generation of JAK inhibitors

**DOI:** 10.31138/mjr.31.1.105

**Published:** 2020-05-25

**Authors:** George Bertsias

**Affiliations:** 1 Rheumatology and Clinical Immunology, University of Crete Medical School and University Hospital of Iraklio, Iraklio, Greece; 2 Institute of Molecular Biology and Biotechnology, Foundation for Research and Technology – Hellas, Iraklio, Greece

**Keywords:** Inflammation, cytokines, growth factors, autoimmune diseases, inflammatory bowel disease, psoriasis, haematological malignancies

## Abstract

Several cytokines and growth factors, as well as their downstream signalling pathways, are implicated in the pathogenesis of haematological and immune-mediated diseases. These mediators act through binding to their cognate receptor and activation of one or more of the four Janus family tyrosine kinases (JAKs). Gene knock-out studies together with evidence from patients carrying activating mutant forms of JAKs (eg, *JAK2* V617F in myeloproliferative disorders) provided strong rationale for the development of JAK inhibitors. Based on encouraging preclinical data showing the capacity of JAK inhibitors to suppress the signalling from multiple cytokines, an extensive drug development program was set out, with the initial successful introduction of tofacitinib, baricitinib and ruxolitinib in various chronic rheumatic and myeloproliferative diseases, respectively. Importantly, advancements with the design of next-generation, hyper-selective JAK inhibitors hold promise for the better control of inflammation, while reducing the risk for harms, in an expanding spectrum of medical disorders.

## INTRODUCTION

Lymphohematopoietic cells are the common denominator in a variety of normal processes and pathologic conditions in humans. To establish and maintain their basal and activated status, differentiation and effector function, these cells receive external signals through soluble mediators including cytokines and growth factors. To name a few examples, erythropoietin (EPO) is the primary erythropoietic factor that acts on bone marrow precursors of red cells to regulate erythropoiesis^1^; interleukin-2 (IL-2) is critical for the maintenance of regulatory T-cells and the differentiation of CD4+ and CD8+ T-cells into effector and memory cells.^2^ Regardless of whether they represent the inciting defect or the end-product of malfunctioning cells, cytokines, as well as their downstream signalling pathways, have been implicated in the pathogenesis of immune- and non-immune-mediated diseases.^3^

In this context, cytokine-targeting treatment strategies have evolved since the last 35 years, initially through the development and marketing of anti-cytokine monoclonal antibodies.^4^ Such treatments, also known as biologic agents or biologic disease-modifying antirheumatic drugs, have proved to be well-tolerated and particularly effective in controlling chronic inflammatory disorders,^5,6^ best exemplified in the case of anti-tumour necrosis factor (TNF) monoclonal antibodies in refractory cases of inflammatory arthritides, psoriatic disease, inflammatory bowel disease and other disorders. Notwithstanding, they are hampered by certain limitations including their narrow mode of action (ie, targeting a single cytokine) and the possible induction of anti-drug (idiotype) antibodies that result in loss of efficacy and/or allergic reactions.^7^

The realization that different cytokines may share common receptor subunits and/or intracellular signalling mediators,^8^ together with advancements in the characterisation of small molecules that penetrate the cell membrane to reach their target, gave an impetus towards the pharmacological development of inhibitors of molecules that are downstream to cytokine receptors, such as Janus kinase (JAK) inhibitors. In this review, a brief description of JAKs and their biology in the blood and immune system is provided, followed by presentation of the milestones in the clinical program development of JAK inhibitors. In conclusion, recent progress with the next-generation, selective JAK inhibitors is discussed, together with future perspectives with regards to innovative applications of this class of agents in an expanding spectrum of clinical indications.

## A BRIEF OVERVIEW OF THE JAK PATHWAY AND ITS RELEVANCE TO HAEMATOPOIESIS AND IMMUNE SYSTEM FUNCTION

Cytokines and growth factors mediate their biological effects through binding to their cognate receptor, which leads to receptor conformational changes (typically, dimerization) and subsequent activation of the Janus family tyrosine kinases (JAKs).^8–10^ Associated JAKs phosphorylate (through their JH1 kinase domain) both each other (autophosphorylation) and the cytoplasmic domain of the cytokine receptor, thus creating a docking site for the signal transducers and activators of transcription (STAT) proteins. Next, JAKs phosphorylate the C-terminus of STATs, which then dissociate from the cytokine receptor, dimerize and enter into the cell nucleus to function as transcription factors binding to chromosomes and tuning the expression of thousands of genes in a tissue–and context–specific manner.^11^ Accordingly, STATs (STAT1–4, 5A, 5B, and 6) are crucial regulators of the activation, survival and maturation/differentiation of immune and hematopoietic progenitor cells but are also implicated in nervous system development.

A total of four different JAKs have been identified (JAK1, JAK2, JAK3, TYK2), and pairs of the same or different JAKs bind to the cytoplasmic domain of distinct receptors, which explains – at least in part – their selective roles and biological functions (*Table 1*).^8–10^ For instance, pairs of JAK2 have been shown to mediate the effects of a number of cytokines and growth factors including IL-3, IL-5, granulocyte-macrophage colony-stimulating factor (GM-CSF), granulocyte-colony stimulating factor (G-CSF), EPO, thrombopoietin (TPO), growth hormone and leptin. In another example, JAK3 is recruited to the γ-common chain (the common subunit of the IL-2, IL-4, IL-7, IL-9, IL-15 and IL-21 receptors) together with JAK1 (i.e., JAK1/JAK3 pair) to transduce the aforementioned signals. An obvious consequence of such signalling architecture is that inhibition of a single JAK member may be capable to abrogate simultaneously the effects of more than one cytokines/soluble factors. Of note, JAK1, JAK2 and TYK2 are ubiquitously expressed, whereas JAK3 expression is restricted to haematopoietic, myeloid and lymphoid cells.^12^

**Table 1.** Cytokines/growth factors, associated Janus kinases (JAKs) and signal transduction and activator of transcription (STATs) molecules.

**Cytokine/growth factor**	**JAK(s)**	**STAT(s)**
Common γ-chain cytokines: IL-2, IL-7, IL-9, IL-15, IL-21	1, 3	3, 5
IL-4	1, 3	6
IL-13	All	6
IL-3, IL-5	2	3, 5, 6
IL-6, 11	1, 2, TYK2	1, 3
IL-12	2, TYK2	4
IL-23	2, TYK2	3, 4
IL-27	1, 2, TYK2	All
GH, Leptin	2	3, 5
EPO	2	5
TPO	2	1, 3, 5
G-CSF	2	5
GM-CSF	2	3, 5
IFN-α/β	1, TYK2	1, 2, 3, 4
IFN-γ	1, TYK2	1
IL-10	1, 2, TYK2	1, 3, 5
IL-19, 20	1, 2, TYK2	3
IL-24	JAK1	3
IL-28, IL-29	1, TYK2	All

The biological effects of JAKs were highlighted through observations in gene knock-out mice and in humans carrying mutant forms of JAKs (reviewed in ^13, 14^). Specifically, JAK1-deficient mice are perinatally lethal with severely compromised lymphopoiesis and unresponsiveness to interferons, γ-common chain cytokines and IL-6. JAK2-deficient mice succumb with defective erythropoiesis due to absent signalling by haematopoietic growth factors (EPO, TPO, IL-3, GM-CSF) and IFN-γ. JAK3-deficient mice manifest severe immunodeficiency as a consequence of blunted γ-common chain cytokines signalling.^15,16^ Similarly, inactivating mutations in JAK3 have been described in patients with severe combined immunodeficiency (SCID) associated with defective T-cell and NK cell function, development, and homeostasis, whereas B-cells are largely spared.^17^ These defects are mostly encountered in individuals carrying two mutated JAK3 alleles; conversely, their heterozygous parents display no overt immune defects which agrees with the clinical observation that partial/reversible inhibition of JAKs may not cause severe generalized immunosuppression. Finally, TYK2-deficient mice are viable with no gross abnormalities, in line with the fact that TYK2, similar to JAK3, is involved in the signalling of a smaller number of cytokines (IFN-γ, IFN-α/β, IL-12, IL-23). Rare cases of human TYK2 deficiency have been reported, associated with increased susceptibility to viral and bacterial infections.^18^

Further insights are obtained through the study of activating mutations in JAKs. A notable example is the V617F (Val617Phe) mutation in JAK2, which is found almost universally in patients with polycythaemia vera, and also in many patients with other myeloproliferative disorders such as essential thrombocythaemia and myelofibrosis.^19^ This somatic mutation causes constitutive JAK2 activation therefore augmenting the signalling events mediated by EPO, TPO and GM-CSF. Also, gain-of-function JAK1 mutations have been reported in adult T-cell precursor acute lymphoblastic leukaemia (T-ALL),^20^ and *JAK3* mutations in a few cases of leukaemia and lymphoma.^21^ More recently, TYK2 mutations were described in T-ALL, particularly the V678F STAT3-activating mutation.^22^ In addition, chromosomal re-arrangements resulting in fusion proteins of JAKs with other transcription factors were found in patients with haematological malignancies. A well-characterized example is translocated E26 transformation specific (ETS) leukaemia (TEL)-Jak2, which comprises the oligomerization domain of ETS family transcription factor (TEL) linked to the JH1 kinase domain of JAK2 and causes unabated activation of STAT3 and STAT5.^13^ Likewise, fusion proteins of TYK2 with other proteins (nucleophosmin 1, polyadenylate binding protein 4, MYB, NF-κB2) have been detected, all associated with aberrant STAT3 activity.^22^

The genetic evidence linking JAKs with autoimmune/inflammatory rheumatic diseases is more circumstantial. To this end, single nucleotide polymorphisms in JAK1 have been associated with juvenile idiopathic arthritis, in JAK2 with Adamantiades-Behçet’s disease, and in TYK2 with systemic lupus erythematosus, inflammatory bowel diseases, psoriasis, systemic sclerosis, and inflammatory myopathies.^23–25^ The exact mechanism by which these genetic variants confer susceptibility remains elusive. Altogether, cumulative genetic, functional and *in vivo* evidence underscores a critical role for JAKs in mediating signals from cytokines and growth factors implicated in haematopoiesis and immune system function under normal and pathologic conditions.

### JAK inhibitors in haematological disorders

A milestone in the history of haematological malignancies was the identification of the inter-chromosomal exchange between chromosomes 9 and 22 leading to the creation of the *Bcr-Abl* fusion gene as the underlying molecular pathophysiology of chronic myelogenous leukaemia (CML).^26^ Notably, *Bcr-Abl* was shown to encode a tyrosine-kinase protein which accounts for the malignant transformation of the myeloid cells. By screening a library for protein kinase C inhibitors, imatinib (also known as Gleevec or STI571) was discovered to inhibit the auto-phosphorylation of *Bcr-Abl*, thus paving the way for the first approved tyrosine kinase inhibitor in medicine.^27^ Indeed, in a succeeding clinical development program, imatinib proved efficacious and safe for all stages of CML, which led to initial marketing approval in 2001. Later, it was found that the compound was not entirely selective for *Bcr-Abl* since it could inhibit also other intra-cellular kinases such as c-KIT (receptor for stem-cell factor) and platelet-derived growth factor receptor (PDGFR).^27^

The successful paradigm with imatinib created expectations for expanding the drug portfolio of tyrosine kinase inhibitors to other malignant disorders. Among the first candidates were other myeloproliferative diseases where the discovery of the activating JAK2 V617F mutation was considered ‘analogous’ to the *Bcr-Abl* translocation in CML.^19^ Notably, a number of other genetic variants/mutations have been linked to cases of *de novo* acute myeloid leukaemia (AML) and myeloproliferative neoplasm-blast phase, which all result in hyperactivated JAK-STAT signalling.^28^

In view of the above, there is strong rationale for therapeutic blockade of JAKs, especially the JAK2 kinase activity and accordingly, a number of JAK inhibitors such as ruxolitinib, TG101348, lestaurtinib and others, are being tested in these diseases. However, as discussed by Srdan Verstovsek,^19^ while *Bcr-Abl* is an oncogenic kinase that does not exist under normal condition, the V617F mutation resides outside the ATP-binding pocket of JAK2 and consequently, any JAK2 inhibitor targeting the ATP-binding pocket may be capable of blocking not only the mutant but also the normal (wild-type) JAK2 kinase. This might have important clinical implications with regards to possible development of myelosuppressive adverse events following treatment with JAK2 inhibitors. Notwithstanding these concerns, several putatively selective JAK2 inhibitors have been developed and tested to treat myeloproliferative diseases. A detailed presentation of the corresponding clinical trials is outside the scope of this review. To this end, ruxolitinib (also known as INCB018424) is an orally administered inhibitor of JAK1 (IC_50_ 3.3 nM) and JAK2 (IC_50_ 2.8 nM), whereas TYK2 and JAK3 are much less affected.^29^ Following supportive evidence in preclinical studies, a number of phase I/II and phase III studies (Controlled Myelofibrosis Study with Oral JAK Inhibitor Treatment [COMFORT-I/II] trials) showed statistically superior effectiveness of ruxolitinib over the “best available treatment” in patients with intermediate or high-risk myelofibrosis, thus leading to the approval of the drug by the Food and Drug Administration (FDA) in 2011.^29^ Ruxolitinib-treated patients exhibited normalization of aberrant STAT3 signalling and reduction in cytokines, angiogenic and fibrogenic factors. No particular safety signal was noted with the exception of frequent (41%) drug dosage modifications due to thrombocytopenia, which may be explained by inhibition of JAK2–mediated thrombopoietin signalling. Trials are underway to determine the efficacy of ruxolitinib in other myeloproliferative diseases, including chronic neutrophilic leukaemia and atypical CML (both often associated with mutations in colony-stimulating factor-3 receptor), as well as AML and T-cell lymphoproliferative disorders.^30, 31^

Lestaurtinib (CEP-701) is a dual JAK2 and Flt3 inhibitor and has been used in Flt3-mutated AML cases yet with no clear signs of effectiveness over standard chemotherapy. Also, in a phase II trial in patients with myelofibrosis, a reduction in spleen size was observed in some patients by week 12, however, gastrointestinal adverse events were very common (50%) and resulted in the study discontinuation.^19, 28, 32^ Pacritinib (SB1518) is another dual JAK2/FLT3 inhibitor that has been approved for the treatment of intermediate- and high-risk myelofibrosis. The compound induces apoptosis in cell lines harboring the JAK2 V617F, FLT3 wild-type and FLT3 internal tandem duplication (FLT3-ITD) mutation, and could potentially overcome FLT3 tyrosine kinase inhibition resistance in FLT3 mutated AML by means of excessive JAK2 inhibition.^19, 28^ The use of pacritinib is T-cell leukemias is also under examination in phase I trials.^31^

Fedratinib (TG101348) is a selective JAK2 inhibitor that triggers apoptosis in cell lines carrying the JAK2 V617F mutation. Importantly, in mice with human JAK2 V617F-induced polycythaemia vera, administration of fedratinib led to improvement in haematocrit, reduction in splenomegaly and prolonged survival. Subsequent clinical program led to approval of the drug for the treatment of myelofibrosis, including ruxolitinib-resistant cases.^33^ Moreover, cytarabine and fedratinib combination therapy has demonstrated efficacy in patients with AML.^28^ Finally, momelotinib (also known as CYT-387) inhibits both JAK1 (IC_50_ 11 nm) and JAK2 (IC_50_ 18 nm), rather than JAK3 (IC_50_ 0.16 μM), although off-target effects (serine/threonine kinase IKBKE) have been described.^19, 28^ The drug was tested in two large phase III trials (SIMPLIFY-1 and -2) in patients with myelofibrosis, with or without prior exposure to ruxolitinib.^34^ Momelotinib failed to demonstrate either non-inferiority against ruxolitinib or superiority over “*best available treatment*” (88% receiving ruxolitinib), although anaemia-related endpoints such as transfusion independence, was significantly improved in the momelotinib arm. Peripheral neuropathy developed in a considerable proportion of momelotinib-treated patients, thus limiting further the potential clinical use of the compound.^29^ Altogether, advancements in our understanding of the molecular pathogenesis of myeloproliferative disorders, with activation of the JAK/STAT pathway being a common denominator, has led to the development of several JAK-targeted therapies with significant effectiveness and balanced efficacy/safety risk ratio.

### JAK inhibitors in rheumatologic disorders

Chronic rheumatologic disorders such as rheumatoid arthritis (RA), spondyloarthropathy/psoriatic arthritis, connective tissue diseases and autoinflammatory disorders, are characterized by perturbed expression and function of inflammatory cytokines. Notably, although in each of the abovementioned diseases, certain cytokines are often considered to play more principal role than others (eg, TNF in inflammatory arthritides, interferon-a in systemic lupus erythematosus), evidence from animal and human studies indicates that a plethora or cascade of cytokines, growth factors and other inflammatory mediators, may contribute to their pathogenesis. This is exemplified in the case of RA, where implicated mediators include interferon (IFN)α, TNF, IL-1, IL-6, IL-7, IL-10, IL-12, IL-15, IL-17, IL-18, IL-21, IL-23, GM-CSF and transforming growth factor (TGF)-β.^35^ Notably, the majority of these inflammatory signals are transduced through various combinations of JAK and STAT proteins. Accordingly, there is rationale for therapeutic targeting of JAKs since this can shut off the effects of multiple involved cytokines and presumably, provided better (or broader-level) control of the rheumatic disease.

Tofacitinib (CP 690,550) was the first JAK inhibitor to be introduced in humans. Pharmacological studies have indicated that the compound blocks JAK3 and JAK1, whereas JAK2 and TYK2 are much less affected; accordingly, common γc chain cytokines, IFN-γ and IL-6 are the main cytokines that are inhibited.^36^ Following encouraging results from preclinical studies in murine and rat models of autoimmune arthritis, tofacitinib entered into clinic. An extended clinical development program including several phase I to III studies established the clinical effectiveness of the drug in active RA, with significant improvements in disease activity, physical functioning, health-related quality of life as well as prevention of bone erosions and structural damage.^37^ Various immunological parameters pertaining to the JAKSTAT signalling pathway and activated peripheral blood immune cell subsets, were also improved.^38^ These data led to approval of the drug, initially by the FDA in 2012, for RA patients who are refractory to or intolerant of methotrexate. Consistent results were noted in controlled trials in psoriatic arthritis, leading to the drug approval during 2017–2018. Concerning psoriasis, although the drug was more efficacious than methotrexate, it failed to demonstrate non-inferiority compared to other biologic agents (anti-TNF, anti-IL12/23, anti-IL17).^37, 39^ Phase III studies are currently underway to determine the drug effectiveness in axial spondyloarthropathy (ankylosing spondylitis). Tofacitinib is also approved for the treatment of active moderate-to-severe ulcerative colitis but not Crohn’s disease.

Baricitinib (LY3009104) is a dual JAK1/JAK2 inhibitor that predominantly suppresses IFN-γ, IL-6, IL-12/23, EPO and GM-CSF signals. It has been approved for the treatment of active RA on the basis of superior efficacy over methotrexate.^40^ Notably, phase III trials have suggested increased clinical responses over anti-TNF agents (adalimumab) as early as 2–4 weeks after treatment initiation. Baricitinib has also yielded encouraging results in psoriasis due to significantly higher clinical improvement (assessed by the PASI75 index) at week 12 as compared to placebo.^37^ Moreover, in a phase II trial of non-severe systemic lupus erythematosus, the drug led to significantly increased clinical responses (assessed by the composite index SRI-4), especially improvements in skin and joint disease, as compared to the placebo arm (standard-of-care).^41^ Ongoing trials will provide definitive evidence regarding baricitinib effectiveness in the above-mentioned, as well as in other diseases, where the drug is also being tested such as atopic dermatitis and giant-cell arteritis.

To this end, cumulative evidence from the clinical development program of these two first-generation JAK inhibitors has yielded interesting insights with regards to their efficacy and safety in rheumatic diseases. Presumably due to their mode of action that involves the simultaneous blockade of several inflammatory cytokines, both tofacitinib and baricitinib show very good clinical responses either as monotherapy or in combination with methotrexate, although the latter provides numerically greater results.^37,39^ The same explanation might account for the tendency for superior efficacy of JAK inhibitors over targeted (anti-TNF) biologics, although this observation warrants further confirmation. Next, the cytokine-blockade effect of JAK inhibitors seems to be dose-dependent and to occur fast after the drug administration, thus resembling the anti-inflammatory effects of glucocorticoids. Nonetheless, *ex vivo* studies in treated RA patients have shown that the inhibition of JAK-STATs is partial and reversible, which explains to some extent, the lack of severe, generalized drug-induced immuno-suppression.^38^ Notably, in spite of the (partially) distinct pharmacodynamic effects of the two compounds (ie, tofacitinib predominantly inhibiting JAK1/JAK3, baricitinib predominantly inhibiting JAK1/JAK2), their safety profile is very similar, suggesting that the actual *in vivo* effects might not be predicted entirely by *in vitro* “selectivity” assays. Thus, treatment with either tofacitinib or baricitinib has been associated with increased risk for cytopenias (albeit of mild degree), viral infections (especially from VZV) and increases in serum lipids, which are all related to JAKs blockade (e.g. viral infections due to dampened IFNα/JAK1 and/or JAK3/NK-cells axis, cytopenias due to dampened JAK3/myelopoiesis axis etc.).^37, 42^

### Second generation of JAK inhibitors

Technological progress in the development of novel pharmacological compounds, coupled with the realization that certain side effects of the first generation JAK inhibitors may be due to non-selective or ‘off-target’ effects, has pushed towards the design and implementation of a new generation, highly selective inhibitors. To date, most efforts in chronic inflammatory diseases have focused on the preferential inhibition of JAK1 and JAK3, since these two JAKs are mostly involved in mediating signals downstream of pathogenic cytokines, while sparing the potentially detrimental effects of JAK2 on haematopoiesis. A few notable examples are listed below.

Upacitinib (ABT 494) represents a selective JAK1 inhibitor that in preliminary trials, has demonstrated efficacy in RA, spondyloarthropathies (psoriatic arthritis, ankylosing spondylitis), juvenile arthritis and inflammatory bowel disease.^37,43^ Filgotinib (GLPG0634) is another JAK1-selective inhibitor which has also shown to be effective in RA, psoriatic arthritis and moderate-to-severe Crohn’s disease.^37,44^ Similarly, itacitinib (INCB039110) inhibits JAK1 much more potently as compared to JAK2/JAK3 and is currently being tested in RA, psoriasis and myelofibrosis.^37,39,45^ Decernotinib (VX-509) demonstrates 5-fold selectivity towards JAK3 as compared with other JAKs and is now being assessed in RA clinical trials.^46^ A selective TYK2 inhibitor, BMS-986165, is being tested in psoriasis, whereas a dual JAK1/TYK2 inhibitor, PF-04965841, is under investigation in systemic lupus erythematosus and psoriasis.^37^ On the other hand, peficitinib (ASP015K) manifests broader spectrum of activity by inhibiting JAK1 but showing moderate effects against JAK3 and TYK2, and only mild effect against JAK2. The drug is now being tested in RA, psoriasis and ulcerative colitis.^47,48^ While preliminary results suggest efficacy of the aforementioned next-generation JAK inhibitors, it remains to be seen how enhanced selectivity will impact on the efficacy/safety ratio.

### Future prospects in therapeutic targeting of JAKs

Considering the implication of inflammatory cytokines and mediators in human diseases, inhibitors of the JAK-STAT pathway are increasingly viewed as plausible therapeutic agents in an expanding spectrum of pathological disorders.^25^ Next to inflammatory arthritides, psoriatic and inflammatory bowel disease, JAK inhibitors are currently being assessed in autoimmune connective tissues diseases such as systemic lupus erythematosus, Sjogren’s syndrome and inflammatory myositis. Inhibitors of TYK2/JAK1 may be particularly effective in the group of genetically defined interferonopathies. Intriguingly, circumstantial evidence suggests that JAK inhibitors may as well be efficacious in a variety of inflammatory or autoinflammatory-spectrum diseases including sarcoidosis, Adamantiades-Behçet’s disease, periodic fever syndromes, giant-cell arteritis, JIA, and adult-onset Still’s disease. In this context, topical drug formulations are also under development thus offering the potential to treat skin and ocular inflammatory disorders.

Another emerging concept in the therapeutic armamentarium of JAK inhibitors is the prospect of administering them in combination with another class of small molecular inhibitor. The rationale to pursue such combinatory approach is based on two main observations. First, despite the extensive anti-cytokine effects of existing JAK inhibitors, still, a proportion of patients with RA or other inflammatory or hematologic diseases may not respond. Second, at the cellular level, the biological effects of JAK inhibitors is highly contextual and depends on the interaction of STATs with other transcription/epigenetic factors and the overall chromatin state.^49^ To broaden the genomic and overall biological effects of JAK inhibitors, strategies have been developed in combining them with other classes of kinases (eg, Bcl-2, Bruton-tyrosine kinase, spleen tyrosine kinase) or with epigenetic modifiers such as histone de-acetylases (HDACs) and bromodomain and extra-terminal domain (BET).^28^ These ‘multi-target’ regimens are currently being explored in haematological malignancies but might as well prove to be useful in rheumatological diseases provided that no strong safety signals arise. For the future, it is foreseen that increasing knowledge about the molecular pathophysiology of haematological and rheumatic diseases, coupled with the post-marketing experience of JAK inhibitors, will help to optimize patient selection and place JAK inhibitors at the right point in the therapeutic algorithm.
